# Cancer Studies under Space Conditions: Finding Answers Abroad

**DOI:** 10.3390/biomedicines10010025

**Published:** 2021-12-23

**Authors:** José Luis Cortés-Sánchez, Jonas Callant, Marcus Krüger, Jayashree Sahana, Armin Kraus, Bjorn Baselet, Manfred Infanger, Sarah Baatout, Daniela Grimm

**Affiliations:** 1 Department of Microgravity and Translational Regenerative Medicine, Otto von Guericke University, 39106 Magdeburg, Germany; jose.cortes@ovgu.de (J.L.C.-S.); marcus.krueger@med.ovgu.de (M.K.); armin.kraus@med.ovgu.de (A.K.); manfred.infanger@med.ovgu.de (M.I.); 2 Radiobiology Unit, Institute for Environment, Health and Safety, Belgian Nuclear Research Centre (SCK CEN), 2400 Mol, Belgium; jonas.callant@ugent.be (J.C.); bjorn.baselet@sckcen.be (B.B.); sarah.baatout@sckcen.be (S.B.); 3 Research Group ‘Magdeburger Arbeitsgemeinschaft für Forschung unter Raumfahrt-und Schwerelosigkeitsbedingungen’ (MARS), Otto von Guericke University, 39106 Magdeburg, Germany; 4 Department of Biomedicine, Aarhus University, 8000 Aarhus, Denmark; jaysaha@biomed.au.dk; 5 Department Molecular Biotechnology, Ghent University, 9000 Ghent, Belgium

**Keywords:** review, gravitation, weightlessness, radiation, neoplasms, gene expression, mechanobiology, gravisensors

## Abstract

In this review article, we discuss the current state of knowledge in cancer research under real and simulated microgravity conditions and point out further research directions in this field. Outer space is an extremely hostile environment for human life, with radiation, microgravity, and vacuum posing significant hazards. Although the risk for cancer in astronauts is not clear, microgravity plays a thought-provoking role in the carcinogenesis of normal and cancer cells, causing such effects as multicellular spheroid formation, cytoskeleton rearrangement, alteration of gene expression and protein synthesis, and apoptosis. Furthermore, deleterious effects of radiation on cells seem to be accentuated under microgravity. Ground-based facilities have been used to study microgravity effects in addition to laborious experiments during parabolic flights or on space stations. Some potential ‘gravisensors’ have already been detected, and further identification of these mechanisms of mechanosensitivity could open up ways for therapeutic influence on cancer growth and apoptosis. These novel findings may help to find new effective cancer treatments and to provide health protection for humans on future long-term spaceflights and exploration of outer space.

## 1. Introduction

Outer space is an extremely hostile environment for human life, with radiation, microgravity, and vacuum posing the most significant hazards. There are numerous other stressors that humans must endure during spaceflights, such as a disrupted circadian rhythm, confined spaces, dietary alterations, and psychological distress.

Microgravity induces bone loss with increased urinary calcium excretion and muscle and heart atrophy, and the otolith-related function also declines. Several studies suggest that the immune system may also be disturbed by spaceflight [1], but there are no reports of astronauts developing serious infectious diseases during spaceflight or after returning. Scientists have studied the carcinogenic effects of radiation and the impact of microgravity (µ*g*) on humans, and the advances in molecular biology, genetics, radiotherapy, and oncology have provided more insights into these matters in recent decades.

In the present review, we focus on the effects of radiation and µ*g* on cancer and discuss the possible countermeasures to their effects. Advances in this area could provide new treatments for life on Earth, where cancer has surpassed heart disease as the primary cause of death in many countries [2].

## 2. Cancer

### 2.1. Definition of Cancer

**Figure 1:**
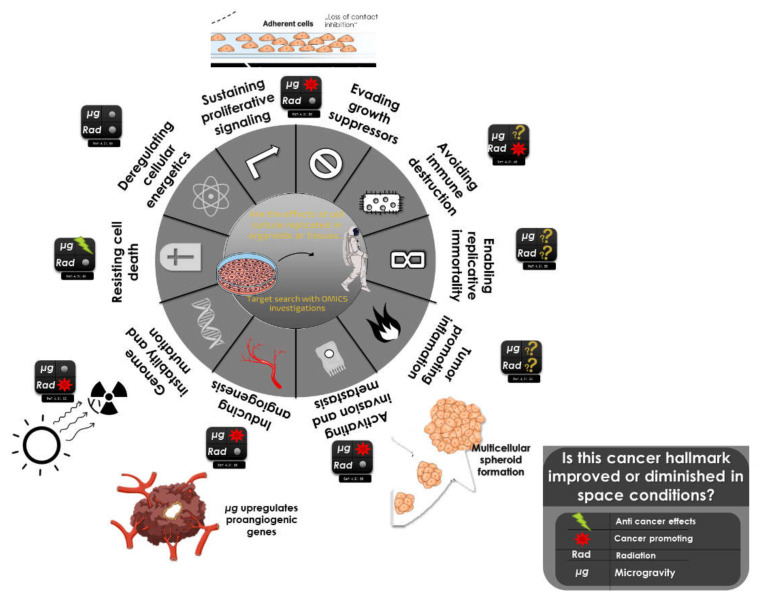
Factors involved in carcinogenesis and promotion of tumour growth.

Cancer is a disease that escapes a straightforward definition. In a broader sense, it refers to more than 277 different types of cancer disease [3]. The model proposed by Hanahan and Weinberg [4] has become seminal in our understanding of cancer and has boosted therapeutic research. The hallmarks of cancer summarise the process of carcinogenesis and cancer progression. Tumourigenesis arises by increased genetic and epigenetic alterations that ultimately convert healthy cells into cancer cells characterised by uncontrolled proliferation, elevated survival, unlimited replicative potential, elevated angiogenic behaviour, and activated invasion potential and metastasis (Figure 1) [5]. DNA repair mechanisms in our cells generally repair the DNA damage that could occur due to cell division or exogenous agents, such as smoke, radiation, or the diet. If the damage is extensive, it activates cell death mechanisms, protecting our body from cancer.

Some of the factors identified in cancer aetiology include DNA mutations that alter protein-coding genes, such as platelet-derived growth factors; components of the insulin-like growth factor axis; transmembrane proteins like RET and NTRK1; sex hormones; tumour-suppressor genes; transcription factors of the SMAD family and the forkhead/winged helix-box transcription factor (Fox) family; and signal transduction pathways, such as sonic hedgehog (SHH), Wnt, and Notch.

Viruses like the human papillomavirus, Epstein–Barr virus, and hepatitis B and C are also involved in carcinogenesis [5]. Recent advances in the field have rendered the previous hallmarks of cancer model outdated, with some authors also considering altered stress response favouring overall survival and the importance of the microenvironment [6].

The National Aeronautics and Space Administration (NASA) has identified the risks that an astronaut will experience in long-term missions in space and has classified the changes as low risk (e.g., microbiome changes), mid-level risk (e.g., telomere loss), and high risk (e.g., neuro-ocular alterations, genomic instability) [7]. Telomere loss and genomic instability are part of the hallmarks of cancer [4].

### 2.2. Epidemiology for Cancer in Astronauts/Cosmonauts

Based on cohort studies from astronauts, space travel did not increase the risk of dying from cancer: standardised mortality rates for cancer were even lower than in the general U.S. population [8]. Astronauts and cosmonauts undergo rigorous selection criteria and medical checks, leading to a healthy worker effect. A more recent study found that compared with professional athletes (to control for the healthy worker effect), data do not support increased mortality for astronauts due to unique exposures received in space [9]. Moreover, there was no increase in mortality from cancer causes in the cosmonaut cohort [10], suggesting that even if ionising radiation impacts the risk of death due to cancer, the effect is not dramatic [11]. It is important to note that, unfortunately, there are only a few epidemiological studies about cancer in the astronaut population. Trying to draw any conclusion frow this should be made with caution, as the costs imposed for spaceflight make it a very narrow field of science, and emphasize the importance of using the different simulated space conditions available on Earth laboratories, to try to understand the cellular and molecular changes of a human organism in space. Moreover, it would be interesting to include astronauts and cosmonauts that undergo long-term missions into deep space because the radiation environment beyond low Earth orbit (LEO) is drastically different.

## 3. General Effects of Microgravity

### 3.1. In Vivo Animal Models

There have not been in vivo studies with active cancer patients or even immunosuppressed animals in space. However, a study on the different organ systems of mice that travelled to space showed that short-term space missions impact the expression in some cancer-related genes in the thymus and spleens (*Casp8*, *Fgfr2*, *Figf*, *Hgf*, *Igf1*, *Itga4*, *Ncam1*, *Pdgfa*, *Pik3r1*, *Serpinb2*, *Sykb*, *Cdc25a*, *E2f1*, *Mmp9* and *Myc*) [12]. In the liver and kidney, there were overexpressed genes related to apoptosis and cell death [13].

### 3.2. Important Considerations for Analysing Cancer Cells in Real or Simulated Microgravity

No human being in history has lacked gravity. Astronauts on the International Space Station (ISS, the gold standard for changes in the human body in µ*g*) are exposed to approximately 90% of Earth’s gravity because the ISS is located approximately 400 km above Earth’s surface. The phenomenon we are studying is the effect of ‘unloading’ conditions on the human body, caused by the perpetual free-fall within an enclosed environment like the ISS. What is the signal that cells are perceiving on Earth and lacking in free fall? It is the reaction force exerted by the surface against which our body is standing, and it is the reason why parabolic flight experiments recapitulate the effect that astronauts feel in µ*g*.

Another critical remark is that the human body is a system, not a single-cell organism. Parabolic flights and space mission results should be interpreted cautiously because a cell plate is not a living organism. Moreover, cell culture flasks have stiffer substrates that allow cells to survive and promote their proliferation, and their conditions do not reflect physiological cellular environments [14]. The results obtained in these flasks do not recapitulate what happens in a tissue, so the results of the unloading conditions generated during experiments make this an extreme comparison, and a physiological stiffness control group is often lacking.

All the forces that cells are sensing are relative to the surrounding environment. The cell in the fingertip does not differentiate if one is standing with their feet over the sea or on top of the hardest structure on Earth. This single cell must only deal with the forces coming from the surrounding structures, mainly transmitted through the musculoskeletal system.

**Figure 2:**
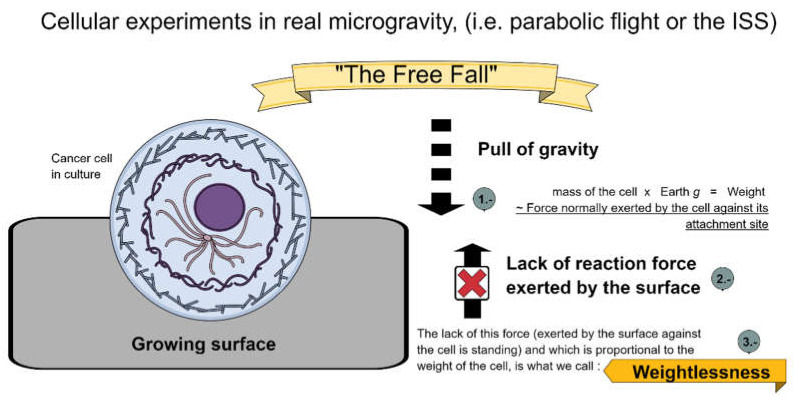
Forces acting in the microgravity environment. A small diagram depicts the weightlessness effect over cells used in microgravity studies and which cells suffer in ISS or parabolic flight experiments: 1.- The mass of the cell exerts a force directly proportional to its mass and the Earth gravity and inversely proportional to its distance to the Earth. 2.- In microgravity conditions, for example in a parabolic flight experiment or inside the International Space Station, the reaction force exerted by the surface against which the subject stands disappears, so the cell is not feeling this physical input anymore. 3.- We call this “weightlessness”, a term that usually appears in microgravity research. Please be aware that other forces like the stiffness of the material’s surface of the flask should not be disregarded. “Dotted”arrow: Direction of the pull of gravity. Arrow with a red X: Direction of the reaction force exerted by the surface, which is lost in microgravity conditions. *g*: Acceleration of gravity.

Figure 2 presents a summary of the forces a cell must deal in “microgravity” conditions.

We will now analyse the most recent literature regarding cancer cells in µ*g* considering this proposed paradigm. We hope that recent advances in mechanobiology will put more order to the contradictory results obtained from cancer cells in µ*g* conditions.

### 3.3. At the Cell Level

µ*g* plays an exciting role in the carcinogenesis of normal and cancer cells, where most of the cells suffer from an increase in the apoptotic rate [15,16], but other cells even gain stemness features [17]. For a more detailed explanation of the results obtained regarding the effect of µ*g* on apoptosis in cells and tissues, please refer to the recent review by Prasad et al. [16]. What happens to cancer cells exposed to µ*g*? A recent review by Chen et al. [18] provides a detailed explanation of this topic. Different results have been observed depending on the cell type and have been recently reviewed in 2017 [19], and more specifically about breast cancer [20] and thyroid cancer [21]. When comparing µ*g* with hypergravity (hyper-*g*) in a suborbital flight experiment with cancer cells, the hyper-*g* stimulus did not cause a substantial effect on cell gene expression [22], and the µ*g* samples indicated that µ*g* is a more potent regulator of gene expression than hyper-*g*.

### 3.4. Multicellular Spheroid Formation

Real microgravity (r-µ*g*) and simulated microgravity (s-µ*g*) environments favour detachment in most cancer cell lines. After a few days exposed to µ*g*, we can easily distinguish two (or even three) different cell populations): the original adherent (AD) cell population on the walls of the flasks, a floating (F) cell population that comes from detachment, and some of these floating cells aggregate to form multicellular spheroids (MCS). The non-adherent cell populations have different gene expression profiles than the adherent cells or the 1 *g* controls.

MCS formation may represent the collective migration effect observed in cancer cells [23]. MCS is a suitable three-dimensional (3D) model of cancer metastasis and shares some features of metastatic cancer cells [24]. Moreover, it may provide insights into cancer biology and progression to help identify new drug/target combinations for future therapies, such as dexamethasone preventing MCS formation [25] and PP2 [26], a Src inhibitor. We can speculate that the dynamic process of focal adhesion formation could be involved in MCS formation.

When MCS emerge in µ*g*, its gene or protein should be compared with that of a non-adherent cell population obtained by a different 3D culture method because some pro-apoptotic pathways will be upregulated by loss of adhesion to the surface. For example, human breast cancer cells grown in 3D culture show reversion of the malignant phenotype [27], results very similar to random position machine (RPM) experiments [20]. Adherence to the substrate is (almost) always mandatory to guarantee cell survival and is a vital input for most cell types. Otherwise, cells will start a proapoptotic programme, except for some cancer cell lines, which are resistant to anoikis [28], allowing them to metastasise. It has been observed in cell lines that come from a metastatic site (for example MCF-7, MDA-MB-231, FTC-133), they easily form MCS once exposed to s-µ*g*, like the RPM [20,21]. Diverse studies have shown that these cell lines have anoikis resistance [29,30,31].

### 3.5. Ground and Space Facilities to Study Microgravity Changes

For r-µ*g* studies, scientists can use parabolic flight experiments (seconds), where the cells go through several cycles of hyper-*g* and r-µ*g*. Sounding rockets (minutes) provide an extended period of r-µ*g* compared with parabolic flights, exposing cells to only one cycle of hyper-*g*. Finally, the ISS (days, weeks) is the most reliable experimental condition, but it is more costly and there are varying degrees of hyper-*g* and vibration during launch and landing to consider.

There are also some ground-based facilities to simulate µ*g*—for example, the tail-suspension rat model for whole organisms, or at the human level, bed rest studies [32]. Moreover, the clinostat, the RPM, the rotary cell culture system (RCCS), and magnetic levitation devices are available at the cell level. These devices are based on rotation, averaging the gravity vector (clinostat, RCCS, and RPM). They have some advantages and disadvantages; for example, in a clinostat, floating cells are often used, and the medium surrounding them equilibrates the sample in the centre of the rotation axis. In an RCCS, cells are suspended in a culture medium and often attached to beads, which affect the cell surface area to which the cells attach and can have some effects. Cells restricted from spreading against extracellular matrices (ECM) become growth-arrested [14]. The clinostat rotates samples in one axis, and various 3D clinostat devices provide two axes of rotation. Among them, the RPM can change the direction and speed of rotation. The medium movement in these devices imposes some fluid dynamics that can cause shear stress over the AD cell population; however, an initial floating cell population can also be used. The RPM produces the greatest shear forces of all methods [18]. Magnetic levitation places the sample between a superconducting magnet that generates a strong magnetic gradient. The different means to simulate µ*g* on Earth is a source of heterogeneity in the studies analysing the effects of µ*g* on cancer cells. For a recent discussion of the different forces a cell is exposed in these devices, see the recent review by Poon [33].

### 3.6. Brief Description of the Biophysics of Cancer in Space

Cell mechanics are the main effects on cells exposed to altered gravity, but little is known about the unique environment of space and its effects on cancer. The mechanical stimuli are an essential input signal for cells, as basic as those mediated by hormones and receptors [34]. The mechanotransduction process comprises (1) force transmission to specialised cell structures; (2) conversion of force into a biochemical signal; (3) and the response of the cell to that signal, which other types of signals can share [34]. Astronauts will be in a hypomagnetic field that is 10,000 times weaker than the geomagnetic field, and the effects of the geomagnetic systems are unknown. Some studies have shown that a hypomagnetic field promotes additional bone loss in mouse femur during mechanical unloading [35], perhaps by inducing iron overload and inhibiting the recovery of µ*g*-induced bone loss [36].

### 3.7. Graviperception System in Non-specialised Mammalian Cells

In µ*g* research, a fundamental question remains unanswered: how do animal cells detect µ*g*? The answer could have profound implications to tackle the alterations the human body experiences in space. Some specialised cell types have a dedicated organelle to measure gravity changes and accelerations, but no results have been obtained from a universal gravity sensor system in mammalian cells. However, is it necessary to have a gravisensor system in cells? Considering that a cell’s forces come only from its surrounding microenvironment, it would not be necessary to have a gravisensor system in every cell. We next introduce a proposed model that summarises the most recent research in gravisensors and mechanobiology, describing the general effects of these changes and how cells coordinate responses to withstand µ*g*.

The tensegrity model is fundamental for analysing the changes that cells experience in µ*g*. Living cells use a tension-dependent form of architecture known as tensegrity to organise and stabilise their cytoskeleton [37]. The cellular tension response differs depending on the pre-stress level (pre-existing tension) in the cytoskeleton, which involves all three cytoskeletal filament systems. Ingber [37] engendered the idea that cells might not need a gravireceptor: ‘Results suggest that gravity sensation may not result from direct activation of any single gravioreceptor molecule. Instead, individual cells in the living organism may experience gravitational forces because of stress-dependent changes in the cell, tissue, or organ structure that alter extracellular matrix mechanics, cell shape, cytoskeletal organisation, or internal pre-stress in the cell-tissue matrix’. This is the basis for understanding the cytoskeleton and membrane fluidity changes in µ*g*.

It has been observed that when gravity increases, the viscosity of the membrane also increases [38], so it becomes less fluid, which could alter the function of most PM-embedded proteins. However, are the membrane fluidity changes the cause or consequence of cytoskeletal tension changes?

#### 3.7.1. The Cytoskeleton Interaction with Microgravity

The cytoskeleton must participate in gravisensing, as it undergoes rapid changes [39] but then adapts. A feedback mechanism must be activated to regain the cytoskeletal contractility. A feedback loop exists where changes in the tension of stress fibres are transmitted to the YAP/TAZ system, which integrates the changes and coordinates a response via upregulation of ARHGAP18, eliciting changes in actin stress fibre tension to reach a new homeostatic state [40].

The mechanosensitivity of cells proceeds as follows. First, small local clustering of integrins contacting the ECM arrange immature focal adhesions on the cytoplasmic side connecting to F-actin. Then, the cells probe the ECM elasticity by using internal tension or contractility via non-muscle myosin on F-actin bundles. When the ECM resistance is high, some proteins, such as Talins/p130ca enable the maturation of focal adhesions with enlarged F-actin bundles. This dynamic process allows a quantitative response of focal adhesions to externally applied forces [34], and the inhibition of any of the elements involved in this process will induce cells to believe they are experiencing a soft ECM even if the ECM is very stiff [14].

In fact, some of the cytoskeletal alterations observed in µ*g* experiments are related to changes in focal adhesion kinases (FAK) [41] and vinculin [42]. Zayzafoon et al. [43] cleverly explained that the first step in these changes might not be the cytoskeleton per se, but rather RhoA, which is deactivated. This cytoskeletal disorganisation is a consequence and not a cause of µ*g*: ‘a model where alterations in the cytoskeleton are a consequence of primary changes of a cytoskeletal remodeler’ [43]. Additionally, in line with these findings, thickening of actin stress fibres occurs in hyper-*g*, and the opposite effect is observed in µ*g* [44]. The actin network recovers after days and, in some studies, even hours [45]. In more recent research, s-µ*g* inhibited focal adhesion in melanoma cells, leading to inhibition of signalling FAK and RhoA [46]. Moreover, in glioma cells, s-µ*g* inhibited FAK, reduced RhoA/Rock signalling and Nek2 expression, and attenuated glioma viability and migration [47].

#### 3.7.2. YAP/TAZ: Mechanosensor Hub and Mechano-Effector

How can rearrangement of the cytoskeleton and its interaction with the extracellular matrix in µ*g* regulate gene expression? We focus on one of the central mechanosensitive regulators of gene transcription, YAP/TAZ, applied to µ*g* studies. However, more regulators discovered by the mechanobiology field have not yet been studied under µ*g* conditions, such as the myocardin-related transcription factor (MRTF) [48]. YAP/TAZ are coactivators of transcription that exhibit cytosol/nuclear shuttling and are central mechanobiological signal integrators [49]. They are central hubs that integrate mechanical, metabolic, extracellular, and intracellular signalling to dictate cell growth, differentiation, and malignancy through specific, context-dependent mechanisms. They have multiple negative regulators in normal tissues and are activated in regenerative or malignant conditions [49].

Below, we present an oversimplification of the mechanical signals that come into this pathway [49]:

- signals from the ECM, mediated by focal adhesions, activate different kinases, like RhoA and Src, depending on ECM stiffness and available area;
- signals from neighbouring cells, by way of tight and adherens junctions, generally downregulate YAP/TAZ nuclear entry by Hippo-dependent and independent mechanisms, which mediate the contact-inhibition process [50];
- polarity in the epithelial cells by the Hippo pathway [51], its primary inhibitor, mainly by phosphorylation and proteasomal degradation, preventing nuclear entry.

Once in the nucleus, YAP binds to members of the TEAD (Transcriptional Enhanced Associate Domain) transcription factors that possess a DNA interaction domain [52]. Some effects are reprogramming cancer cells into a more malignant phenotype promoting stemness, chemotherapy resistance, and metastases. The primary regulation of YAP activity is its nuclear-cytoplasmic ratio, mediated mainly by the nuclear export of YAP and not the changes in gene/protein level of YAP [53]. In addition, force triggers YAP nuclear entry by regulating transport across nuclear pores [54]. Once the nuclear concentration of YAP has increased, its nuclear activity increases the transcription of genes involved in focal adhesion complex formation. The result is a positive loop, where an increased YAP nuclear-cytoplasmic ratio promotes more focal adhesions, leading to more YAP nuclear localisation [55]. An example of applying mechanobiology to µ*g* research showed that the changes in nuclear shape and gene expression observed in s-µ*g* are dependent on the Linker of Nucleoskeleton and Cytoskeleton (LINC) complex [56].

Researchers showed that YAP is essential for 3D organ homeostasis withstanding gravity [57]. Another group showed that YAP nuclear localisation decreased in the s-µ*g* group of the musculoskeletal cells compared with the 1 *g* control [58], and low-intensity vibration restored their nuclear levels. These data point to a new kind of treatment for the bone and muscle loss observed in µ*g*. These findings could be explained because of the unloading conditions generated by µ*g*. In line with these findings, the depolymerised actin cytoskeleton in s-µ*g* inhibits osteogenic differentiation of bone mesenchymal stem cells by impeding nuclear aggregation of TAZ [59]. Stabilising the actin cytoskeleton with the drug jasplakinolide significantly restored TAZ nuclear levels even in µ*g* conditions, and lysophosphatidic acid had the same positive effects.

#### 3.7.3. Coherent Model: Mechanobiology and Cancer in Microgravity

Based on the published studies, we propose the following system:

(1) Integrins, as anchors of the cell to the extracellular matrix, are constantly sensing and measuring the ECM stiffness (forces generated by the layer against the cell is anchored).

(2) Once µ*g* creates unloading conditions in the cell flask, the force generated by the flask against the cells is lost (because now the flask is in free fall, and there is no reaction force from the surface), and only the force generated by the stiffness of the material of the plate is present.

(3) The cytoskeleton suffers tremendous disorganisation, explained by the tensegrity model. The tension transmitted through the actin stress fibres is lost.

(4) As part of the tensegrity model, the fluidity of the plasma membrane changes in response to the lost tension, and the function of proteins attached to the plasma membrane is altered because of fluidity changes.

(5) The signals of the cytoskeletal disorganisation arrive at the nucleus by pathways like the hub mechanosensor coactivators YAP/TAZ.

(6) YAP/TAZ are also mechano-effectors and coordinate a countermeasure response to adapt the gene expression to the new tensional state of the cytoskeleton, for example, by upregulating genes from the ARHGAP/ARHGEF family, that can regulate Rho GTPase activity. In this way, the cytoskeleton regains its organisation.

(7) An open question in this process is whether the cell can also adapt its plasma membrane lipid content to counteract the fluidity changes.

**Figure 3:**
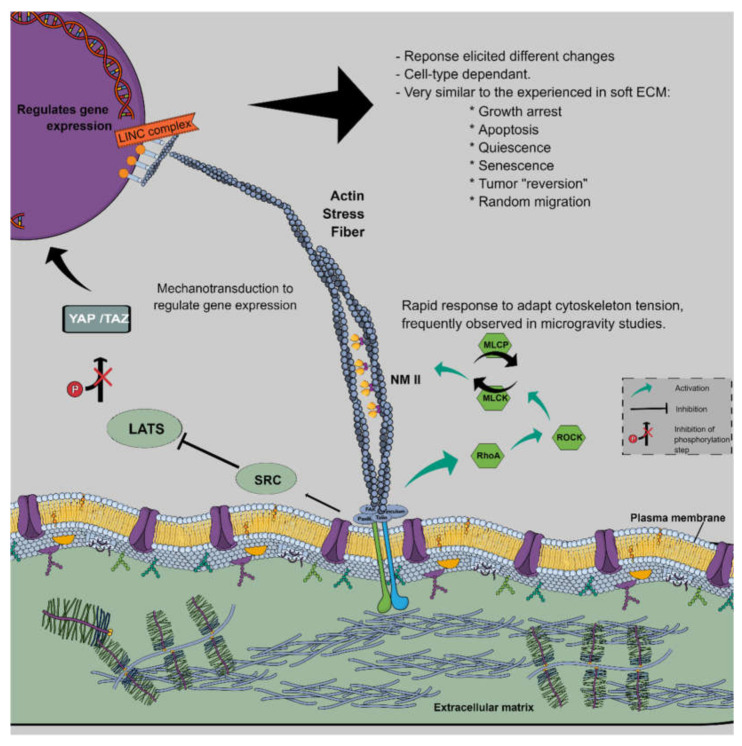
Model for gravisensing in non-specialised mammalian cells: Model for stiffness sensing adapted to µ*g*-studies in cell cultures; any disturbance in this mechanotransduction process will trick the cell into thinking that it is in a µ*g*-environment or, what could be similarly interpreted, a soft ECM. Different mechanotransduction mechanisms presented: (1) Direct force transmission through focal adhesions to organelles, (2) The regulation of mechanoresponsive transcription factor complexes (we show only YAP/TAZ, but others like MTRF are also important). In addition, a vital mechanotransduction process occurs through mechanically gated ion channels, like Piezo1/2, which is related to the tension of the plasma membrane. Responses to soft ECM adapted from [58]. ECM: Extracellular Matrix; LATS: Large Tumour Suppressor; MLCK: Myosin light-chain kinase; MLCP: Myosin light-chain phosphatase; NMII: Non muscle myosin II; ROCK: Rho-associated protein kinase; SRC: Proto-oncogene tyrosine-protein kinase Src; YAP: yes-associated protein. Figure created in the Mind the Graph platform.

Bauer et al. [60] have confirmed the critical role of focal adhesions: FTC-133 cells growing in monolayers or MCS after RPM exposure incorporate vinculin, paxillin, FAK1, and adenine diphosphate (ADP)-ribosylation factor 6 in different ways into the focal adhesion complex. The recent discoveries that link the changes in mechanotransduction to the regulation of metabolism could explain some of the results observed in the µ*g* cellular studies, and this area is growing fast, which will have profound implications. For example, there is mechanosensitive regulation of intracellular pH, in which increased adhesion forces increase the efficiency of glycolytic metabolism, sustaining cell proliferation in firmly attached cells (Figure 3) [61].

## 4. General Effects of Radiation on DNA/Cancer Cells

**Figure 4:**
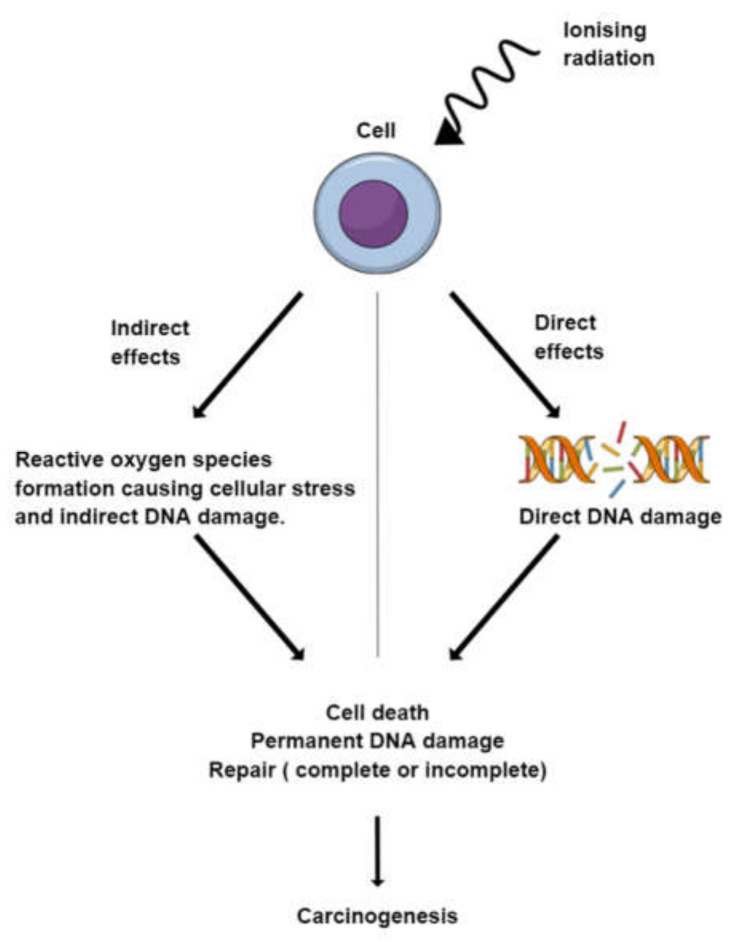
Summary of the effects of radiation on cells. DNA: Deoxyribonucleic acid (Adapted from [62]).

Ionising radiation can cause extensive damage to the DNA inside our cells. Following irradiation, our cells detect DNA damage and either go into apoptosis if the damage is extensive enough or halt the cell cycle and repair the damage. The main pathways through which this damage is repaired are the base excision repair (BER), non-homologous end-joining (NHEJ), and homologous recombination (HR), each of which focusses on the repair of different types of DNA damage. These repair mechanisms, however, are not without fault, as they can introduce and cement errors into our DNA. These errors can be potentially carcinogenic if they occur in a tumour-suppressor gene or an oncogene. Figure 4 provides a summary of the effects of radiation on cells.

### 4.1. High Versus Low Linear Energy Transfer

Numerous studies have shown that high-linear energy transfer (LET) radiation is more damaging than low-LET radiation because the latter produces more complex and hard-to-repair DNA damage [63,64,65,66,67,68,69,70,71,72,73,74,75]. While less complex DNA lesions can easily be repaired by our DNA damage repair mechanisms, this complex DNA damage bogs down these repair mechanisms and induces frequent errors and mis-repairs. The cell can then either go into apoptosis due to accumulated DNA damage or continue through the cell cycle, cementing this DNA damage.

The differences and interactions between high-LET and low-LET radiation are rather complex. However, some deductions can be made. High-LET radiation is more damaging because it produces more complex DNA damage. While the DNA damage repair mechanisms can repair the less complex DNA damage caused by low-LET radiation, these complex DNA lesions take more time to repair, bogging down the repair mechanisms while inducing frequent errors during the repair. This leads to increased apoptosis, or the cell goes through its cell cycle, chromosome aberrations, and cumulative DNA damage [63,64,66,67,69,70,71,72,73,76]. This accumulation of DNA damage, in turn, causes higher incidences and grades of cancer, mainly if these DNA lesions occur in critical genes, such as tumour-suppressor genes or oncogenes [70]. Researchers had thought that the radiosensitivity of cells is directly correlated to the turn-over of these cells, which would cause regenerative tissues and undifferentiated stem cells in active mitosis to be the most sensitive to radiation [77,78]. However, Bielefeldt-Ohmann et al. [74,75] and Weil et al. [74,75] have shown that exposure to high-LET radiation does not increase the incidence of leukaemia, but it does increase the incidence of hepatocellular carcinoma. The elevated relative biological effectiveness (RBE) of high-LET radiation causes sufficient damage in a radiosensitive, high-turnover tissue to cause the cells to go into apoptosis, preventing malignancy.

By contrast, less radiosensitive tissues have time to engage the repair mechanisms, either repairing the damage or introducing mis-repaired sequences and leading to the accumulation of DNA damage and carcinogenesis. Furthermore, as most of the studies have been performed for a short period of time, there is limited data on how different types of radiation could cause different disease progression. Do tumours caused by high-LET radiation have a more aggressive phenotype than tumours caused by low-LET radiation, causing a more rapid disease progression? Further research comparing the effects of low-LET and high-LET radiation on different tissues with varying radiosensitivity is required, alongside long-term studies regarding cancer progression. We do not yet fully understand why there is such variation in cancer development for different tissues and radiation types.

### 4.2. Mixed Beam Radiation and Sequential Exposure

In addition to the differences between high-LET and low-LET radiation, there are conflicting results on whether irradiation of cells with low-LET radiation before high-LET radiation, as would occur in a space environment, has a synergistic or protective effect on DNA damage. Most researchers have stated there is a detrimental effect of pre-irradiation with low-LET radiation followed by high-LET radiation, with peak chromosomal damage at a 30-min interval between the irradiation regimes [68,79,80,81,82,83,84,85,86]. However, Buonanno et al. [87,88] and Stoilov et al. [87,88] have observed a protective effect if the interval is up to 24 h, with researchers attributing this effect to the upregulation of redox scavengers of antioxidants. It is speculated that a short time interval leads to the accumulation of DNA damage, causing more complex lesions, while the repair mechanisms have insufficient time to repair the initial damage. Moreover, a short interval could mean the cells do not have the time needed to upregulate the DNA damage repair mechanism pathways or produce the proteins and molecules needed for redox scavenging [68,89].

### 4.3. Indirect Damage, Non-Targeted Effects, and Bystander Effects

In a review conducted by Barcellos-Hoff et al. [90], the authors hypothesised that, in addition to the direct targeted effects of radiation, there must be multicellular interactions and alterations in the microenvironment that enable carcinogenesis and neoplastic progression. This was confirmed in a study that seeded oncogenically primed cells into healthy and irradiated tissues, respectively. These seeded cells gave rise to rapidly growing tumours in the irradiated tissues but not in the healthy tissues, hinting at the poorly understood non-targeted effects (NTE) [90]. Subsequently, Mavragani et al. [91] presented the idea that ionising radiation produces continuous inflammatory reactions, releasing inflammatory cytokines and producing reactive oxygen species (ROS), further damaging the irradiated cells and their immediate environment. Hada et al. [89] highlighted that one such component of intercellular signalling is nitric oxide (NO), as human fibroblast cells irradiated with iron ion particles and treated with NO scavengers in the culture medium had significantly fewer chromosomal aberrations than irradiated cells without added NO scavengers [89]. Beheshti et al. [92] showed that FYN is upregulated in murine cardiomyocytes and human umbilical cord endothelial cells. FYN upregulation reduces ROS, diminishing the indirect damage of ionising radiation [92]. Furthermore, Liu and Reiter [93] found that exposure of neurons to iron ion particle radiation leads to increased ROS levels that can be counteracted by melatonin treatment. This treatment also increased the number of immature neurons and proliferating cells in mice, which is a sign of an enhanced regenerative capacity of the neurons [93].

Indeed, recent studies point towards an essential role of bystander effects and NTE, further increasing the RBE and carcinogenic potential of high-LET radiation [94,95]. Ionising radiation produces ROS in cells and the extracellular environment, and several authors have discovered an upregulation of ROS scavengers and an increase in intercellular signalling of inflammation following irradiation. However, these changes can be counteracted by administering redox scavengers or antioxidants, effectively reducing the amount of ROS in the extracellular environment and possibly reducing intercellular inflammatory signalling, hinting at the possibility of using pharmaceutical protection against radiation-induced damage [89,90,91,92,93].

### 4.4. Accurate Space Radiation Simulation

In most studies, researchers have used single or double radiation beams with fixed energies, while galactic cosmic rays (GCR) represent a spectrum of radiation and energy. Therefore, the simulations here on Earth are not entirely representative of the radiation environment in outer space. Accurate models of GCR and the radiation environment in LEO, on the lunar surface, or the Martian surface already exist, yet our abilities to mimic or simulate this radiation lag behind. Recent advances with dual-beam irradiation are a step in the right direction, but much work remains to simulate the radiation environment in outer space [68,79,86,96].

### 4.5. Low-Dose Radiation

To accurately evaluate the effects of radiation in a short-term study setup, most of the research has been done with moderate to high radiation doses of several cGy up to several Gy. However, little is known about the effects of low-dose radiation (<100 mGy). The effects of radiation have long been believed to have a linear relationship with excess cancer risk. However, this has not been established for low-dose radiation [97,98]. A statistically significant increase in cancer induction has hardly been described with doses <100 mGy, even in cohort studies following atomic bomb survivors or following the Chernobyl disaster [97,98].

The problem here is that the carcinogenic effect of low-dose radiation requires a sufficient sample size to determine a significant increase in cancer rate. As Ali et al. [98] stated: ‘if excess cancer death cases have been recorded in a sample size of 500 persons in response to 1000 mGy dose exposure, then a sample size of 50,000 would be needed for documenting the carcinogenic effect of 100 mGy, and ≈5 million for ten mGy dose. In other words, the sample size should increase as the inverse square of the dose in order to maintain the statistical precision and power.’ We do not yet have valuable information because it is estimated that an astronaut on a mission to Mars might exceed up to 1 Gy of radiation over 2.5 years (or 1.1 mGy per day). Moreover, it is unknown if the bystander effects and the protective effect of prior low-LET irradiation are also true for chronic, low-dose irradiation [98,99].

### 4.6. DNA Repair Pathways and Markers under Space Conditions

Various research has analysed the effects of microgravity and radiation on the DNA repair machinery. A complete review of this matter can be found in the manuscript of Villanueva et al. [100]. One component of the DNA repair machinery that has been shown to be affected in space are the cell cycle regulator genes. By making use of a 3D clinostat synchronized to a carbon ion or X-ray irradiation system, Ikeda et al. found that the expression of cell cycle-suppressing genes decreased and that of cell cycle-promoting genes increased after carbon-ion irradiation under μ*g*. As a consequence, irradiated cells under µ*g* may pass through cell cycle checkpoints even with DNA damage, suggesting increased genomic instability in space [101]. A similar experimental setup analysed the repair kinetics inside lymphocytes via the measurement of γH2AX foci, a marker of DSB DNA damage. Results showed that incubation in s-µ*g* conditions during DNA repair delayed the rate of radiation-induced DSB rejoining [102]. By exposing cells to s-µg after irradiation, Mognato et al. found that these conditions did not significantly change the expression of genes involved in DNA repair (interestingly, the genes for NER and BER were upregulated), suggesting that transcriptional impairment is not responsible for the increase of mutant frequency [103].

Another explanation of genomic instability in space conditions could be found in a disturbed DNA damage response and repair mechanisms. One study found that s-µ*g* promotes the apoptotic response through p53/PCNA and ATM/ATR-Chk1/2- controlled DNA damage response pathways. These changes could leave the cell more susceptible to DNA damage caused by ionizing radiation exposure [104]. A more recent paper showed many molecular disturbances caused by s-µ*g* in the DNA damage response: s-µ*g* induces both DNA damage as well as the differential expression of DNA repair genes, altering the overall DNA repair capacity and resulting in the activation of ATM/ATR-Chk1/2, Ku70/80 and DNA-PK which regulate NHEJ and HR [105].

An obstacle for determining DNA damage repair kinetics in space is the multitude of detection methods that exist. One of the most used assays to examine DNA damage detect the presence of γH2AX foci, part of DNA DSB foci. For example, by using γH2AX foci detection it was found that s-µ*g* combined with radiation exposure induced DNA DSB formation in an additive manner [106]. Other markers commonly used are chromosomal aberration (CA) frequencies. For example, in cultured human lymphoblastic TK6 cells exposed to X-ray or carbon ion radiation and simulated s-μ*g* conditions, a higher frequency of both simple and complex CA types were observed when cells were exposed to both stressors simultaneously in comparison to cells exposed only to radiation [107]. Furthermore, other commonly used assays are the HPRT mutant frequency analysis and the micronucleus assay With the latter it was for example found that fibroblast irradiated in s-µ*g* conditions had a reduced amount of apoptosis accompanied by an increased fraction of damaged cells [108].

To protect human cells from radiation exposure under space conditions, many methods are being studied. Along with shielding, which has not been thoroughly studied with human cells, the use of radioprotective drugs or agents has been suggested. In some of the studies previously mentioned, compounds, such as nitric oxide scavengers and melatonin have been shown to reduce the adverse effects of radiation. Furthermore, vitamin C, vitamin E, and ascorbic acid have been examined as radioprotective compounds [89]. However, the problem with these countermeasures is that all of them have been studied in a high-dose radiation environment, making them unproven to work in a low-dose environment, such as space [109]. A more detailed discussion can be found elsewhere [110].

## 5. Combination of Radiation and Microgravity

Space conditions expose astronauts to both radiation and µ*g* at the same time, but little is known about the interaction of both stressors in normal cells. Are the dangerous effects observed with radiation and µ*g* synergistic? We present the crucial literature on this topic, first with cellular studies and then whole organism studies and research in real-space conditions.

For lymphocytes growing in a rotating-wall vessel (RWV), activation- and radiation-induced apoptosis is inhibited compared with stationary conditions in a plastic flask, suggesting that cells become more resistant to radiation damage in an s-µ*g* environment [111].

During an 8-day Space Shuttle flight, lymphocytes of a crewmember were exposed to up to 3 Gy gamma radiation before and after the mission; no changes in DNA damage were found. Moreover, for cultured human colon cancer cells on this mission, there were more mutations in the cells from the mission compared with ground controls [112,113].

Lymphoblasts irradiated with gamma rays and subsequently cultured in an RWV for 24 h showed reduced apoptosis in the s-µ*g* group and more damaged cells, as measured by HPRT mutant frequency [108]. Similarly, incubation in s-µ*g* after irradiation did not significantly change the expression of genes involved in DNA repair, suggesting that transcriptional impairment was not responsible for the increased mutant frequency observed in irradiated cells incubated in µ*g* compared with the 1 *g* control [103]. Analysing the repair kinetics inside the lymphocytes via the measurement of gamma-H2AX foci (a marker of double-stranded break DNA damage) demonstrated a slower repair of DSB in cells cultured in s-µ*g* after gamma irradiation [102].

S-µ*g* can affect the DNA-damage response to ionising radiation in peripheral blood lymphocytes (PBL). Exposure to cells in an RPM to s-µ*g* leads to altered expression of some microRNA (miRNA) molecules of irradiated PBL. let-7i*, miR-7, miR-7-1*, miR-27a, miR-144, miR-200a, miR-598, and miR-650 are deregulated by the combined action of radiation and µ*g*. These miRNA–mRNA interactions translate into altered DNA damage repair kinetics, slowing the repair of radiation-induced DNA damage [114].

B lymphoblasts exposed to carbon ion radiation in an RWV showed an increased apoptotic rate, reduced cell survival, and amplified intracellular ROS generation. This induced ROS-sensitive extracellular signal-regulated kinase (ERK)/mitogen-activated protein kinase phosphatase 1 (MKP-1)/caspase-3 activation pathway, promoting cell death, and the upregulation of MKP-1 was insufficient to inhibit the increase in apoptosis. ROS scavengers, such as *N*-acetylcysteine and quercetin reversed these phenomena [115].

In another study, researchers examined the effects of continuous low-dose radiation (high-LET neutrons and low-LET gamma rays) in the presence or absence of s-µ*g* (through incubation in an RPM) on murine fibroblasts for 65 h. Low-dose radiation downregulated genes involved in cytoskeletal remodelling, DNA damage response pathways, and cell cycle regulation. Exposure to s-µ*g* induced oxidative stress response genes and decreased the expression of genes involved in cytoskeletal remodelling, possibly through the Rho signalling pathway. Genes or gene sets altered in the individual treatments were not altered in the combined treatment, indicating a complex interaction [116].

Murine neurons exposed to either acute X-rays of 0.1 or 1.0 Gy or chronic mixed neutron and X-ray irradiation at 0.2 mGy per day for 5 days and RPM showed that radiation alone had the least pronounced effect on the neurons, temporarily stalling the growth of neurites, while only the highest dose impaired survival. Exposure to s-µ*g* and a combination of s-µ*g* and radiation led to the most potent effects on survival and morphology, and chronic low-dose exposure led to no noticeable morphological changes. Gene expression analysis showed changes in genes related to synaptic communication, cell survival, and neurite extension, which indicate that combined exposure reduces neuronal network integrity and survival [117].

The DNA damage response and cytokine production of immune cells were analysed under the combined effects of incubation in an RWV and radiation, alongside the presence of stress hormones (which could contribute to the effects observed on astronauts because stress hormones influence cell proliferation, apoptosis, DNA repair, and T cell activation). Peripheral blood mononuclear cells were treated with isoproterenol and exposed to gamma rays at 0.8 or 2 Gy while incubated in an RWV. There were synergistic effects on the expression of the β2-adrenergic receptor gene, and cells incubated in µ*g* had more DNA strand breaks compared with normal gravity. Moreover, radiation-induced cytokine production occurred only in µ*g*, and isoproterenol prevented most of the effects mediated by s-µ*g* [118].

Human fibroblasts were exposed to either X-ray or carbon ion irradiation of up to 1.5 Gy during s-µ*g* through a new method that did not require stopping the RPM for irradiation. Cells exposed to irradiation and s-μ*g* showed a higher frequency of both simple and complex types of chromosomal aberrations compared with cells irradiated under the static condition by either X-rays or carbon ions. Cell survival was the same for 0.5 Gy of carbon ion irradiation and 1.5 Gy of X-ray irradiation, but the cells exposed to carbon ion radiation had 2–3 times more chromosomal aberrations [119].

In fibroblasts exposed to 1 Gy of carbon ion or X-ray irradiation cultured in an RPM for 48 h, RNA sequencing results showed that the expression of cell cycle–suppressing genes decreased and the expression cell cycle–promoting genes increased after s-µ*g* and carbon ion irradiation. This result suggests increased genomic instability in the space environment, as these cells do not undergo cell cycle arrest to repair DNA damage [101].

Human lymphoblastic cells exposed to up to 1.5 Gy of X-ray or 0.5 Gy of carbon ion irradiation and s-µ*g* through an RPM for 24 h presented a higher frequency of both simple and complex types of chromosomal aberrations in cells exposed to both factors compared to cells exposed to radiation only. These results confirm that carbon ion irradiation produces more DNA damage than X-ray irradiation [107].

In human lung bronchial epithelial cells cultured on a 3D clinostat and irradiated with 2 Gy of X-ray irradiation, the combined effect induced a decrease in the survival fraction, proliferation inhibition, apoptosis, and DNA double-stranded breaks as an additive effect. It also induced RAC2 upregulation, leading to increased intracellular ROS yield from NADPH activity [106].

In a whole organism experiment, mice exposed to s-µ*g* through hind-leg unloading (HLU) and up to 2 Gy of carbon ion irradiation presented an increase in apoptosis and DNA damage in spermatogenic cells in the suspension group. This effect was compounded by radiation in a dose-dependent manner [120].

In a study of mice being suspended by HLU and exposed to low-dose, low-dose-rate gamma irradiation of 0.04 Gy over 21 days, the level of an oxidative specific marker for lipid peroxidation was significantly elevated in the cortex and hippocampus. Moreover, the highest level of nicotinamide adenine dinucleotide phosphate oxidase 2 (NOX2) expression and the lowest superoxide dismutase (SOD) expression and microvessel densities were found in the combination group [121].

An exciting study compared actual space conditions on the ISS using fibroblasts, treated with bleomycin to induce DNA damage, in confluent and exponential growth conditions. The researchers found that the proliferation rate between flight and the ground might be responsible for the shift in the observed DNA damage patterns. A qualitative comparison of the responsive pathways between the flown and ground cells showed a similar microarray analysis of gene expression [122].

In another experiment, mice were subjected to HLU for 7 days and then exposed to 50 cGy of radiation to simulate GCR and sizeable solar particle events, and then HLU for another 7 days. Radiation alone or with HLU increased apoptosis of endothelial cells. Endothelial nitric oxide synthase (eNOS) levels were significantly elevated in the retina after irradiation only or combined with HLU at day 30 after treatment, and the most robust changes were observed in the combination group. For haematological parameters, the main effects for time and radiation were on day 4 after treatment, with a small effect on HLU. This result suggests an early effect of low-dose radiation and spaceflight conditions on the retina and immune cell populations [123].

The recent development of a clinostat that allows s-µ*g* and radiation to be delivered simultaneously should be helpful for future studies. Moreover, whole organism studies are less biased than cell culture plates. We can again observe pronounced differences in the studies that compare 1 *g* controls with s-µ*g* experiments. However, in whole organism studies, the effects are less pronounced and more enjoyable to analyse. For example, Lu et al. [122] showed similar percentages of types of DNA damage patterns between flight and ground cells. The differences observed between the whole organism and cell culture studies make us consider the extent to which the results obtained in the s-µ*g* devices are a consequence of the bias factor intrinsic to these devices. Apart from the interplay between µ*g* and radiation, other stressors associated with an outer space mission have not been studied extensively, such as immune changes, psychological stress, or sleep disturbances, which have already been associated with a higher risk of cancer development on Earth [95]. More research is necessary for a complete understanding of carcinogenesis in space.

## 6. Updated Knowledge on Microgravity Research

Building on the previous knowledge about the effects of µ*g* on cancer and tumour cell processes [19], we next describe the latest relevant research from the last five years regarding cancer cells exposed to r-µ*g* or s-µ*g*. 

### 6.1. Breast Cancer

#### 6.1.1. Real Microgravity Studies

MCF-7 cancer cells exposed to r-µ*g* for brief periods through means of a sounding rocket (6 min) or several parabolic flights (22 s) had reduced E-cadherin protein synthesis.

**Figure 5:**
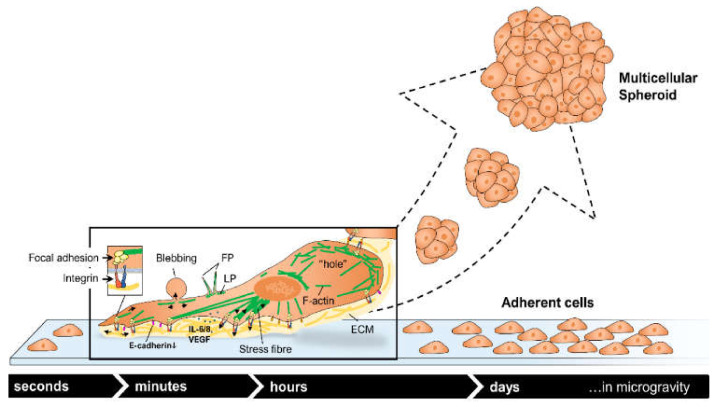
The effects of microgravity on cancer cells. FP: filopodia; LP: lamellipodia; ECM: extracellular matrix; VEGF: Vascular Endothelial Growth Factor. (The figure was originally published by our group in Nassef et al. [20]).

The gene expression of *KRT8*, *RDX*, *TIMP1*, and *CXCL8* was upregulated early, with downregulation of *VCL*. Live-cell imaging showed extensive cytoskeletal rearrangement through alterations of F-actin and tubulin with holes, accumulations in the tubulin network, and the appearance of lamellipodia-like (LP) and filopodia-like (FP) structures [124]. Figure 5 provides a summary of these changes.

MDA-MB-231 triple-negative breast cancer cells were subjected to s-µ*g* and r-µ*g* and the effects of vibration and hyper-*g* to simulate the different stages of a parabolic flight. After 2 h of s-µ*g*, hyper-*g*, or vibration, there were no signs of apoptosis in the cells. The parabolic flight manoeuvres induced early upregulation of *ICAM1*, *CD44*, and *ERK1* mRNA and delayed upregulation of *NFKB1*, *NFKBIA*, *NFKBIB*, and *FAK1* after the last parabola. ICAM-1, VCAM-1, and CD44 protein levels were elevated, whereas the NF-κB subunit p65 and annexin-A2 protein levels were reduced after the 31^st^ parabola.

Vibration did not affect the cells. Genes encoding NF-κB components were upregulated in cells exposed to r-µ*g*. However, the expression of NF-κB-inhibiting proteins increased in the r-µ*g* and the hyper-*g* cultures, possibly explaining the discrepancy between the upregulated NF-κB genes and the normal proteins amounts.

CD44 was upregulated in the r-µ*g* and hyper-*g* cell cultures. CD44 and NFKBIA were upregulated in the hyper-*g* culture, showing that the hyper-*g*-phase of the parabolic flight seems to influence both. The µ*g* phase is the driving factor of most gene expression changes for all other genes [20,125].

#### 6.1.2. Simulated Microgravity Studies

Breast adenocarcinoma MCF-7 cells exposed to s-µ*g* for 24 h, using an RPM, formed AD cells in a monolayer or MCS. Gene array technology showed differences in the expression of genes affected by the oxygen level as well as genes that regulate glycolysis. There was significant upregulation of the expression of enzymes that degrade haem, *ANXA1*, *ANXA2*, *CTGF*, *CAV2*, *ICAM1*, *FAS*, *Casp8*, *BAX*, p53, *CYC1*, and *PARP1* in MCS compared with the 1*g* control and AD cells. Genes related to iron metabolism were generally upregulated during ferroptosis [126], and cell–cell contacts formed in epithelial cell monolayers enhance the resistance to ferroptosis induction. The upregulation of apoptosis, denoted by increased *P53*, *CYC1*, *PARP1*, *FAS*, *CASP8*, and *ANXA1* expression, may be explained by the loss of surface adherence, a vital stimulus for cell survival [127]. NF-κB was mainly localised in the nucleus of MCS, and it may play a role in the MCS formation. PARP-1 is associated with a poor outcome in breast cancer and was markedly upregulated in the MCS, but treatment with a PARP-1 inhibitor did not interfere with MCS formation [128].

AD and MCS populations of MCF-7 cells exposed for 14 days to the RPM showed different changes [26]. MCS showed a significant decrease in E-cadherin protein expression, which plays a role in the cell–cell adhesions. Proteins of the E-cadherin autodegradation pathway were enhanced, and c-Src was detected. PP2 prevented spheroid formation, a tyrosine kinase inhibitor (mainly c-Src), and the E-cadherin antibodies did not suppress MCS formation.

In MDA-DB-231 breast cancer cells cultured in an RWV for 7 days, there were more apoptotic cells in the s-µ*g* group, as revealed by flow cytometry. Electron microscopy showed more secondary lysosomes in the µ*g* group, alongside reduced expression of Bcl-2, which plays an essential role in inhibiting apoptosis. The proportion of cells in the S phase was increased and the expression of cyclin D3 was elevated, consistently with the cell cycle changes. The migration capacity decreased in the µ*g* culture, with a corresponding decrease in the expression of matrix metalloproteinase-9 (MMP-9), a proteinase involved in metastasis [129].

Adenocarcinoma CRL-2351 breast cancer cells (negative for oestrogen receptors, overexpress the HER2/neu oncogene) formed MCS after 24 h of s-µ*g* in an RPM. The AD cells showed increased *BRCA1* and *VCAM1* expression and decreased *KRAS* and *VIM* expression. MCS showed increased expression of *VCAM1* and decreased expression of *VIM*, which could mean that s-µ*g* alters cell repair and adhesion properties. These findings were later analysed at the protein level. In this short-term study, the *VIM* gene was downregulated in the AD cell population, the opposite of what had been observed in the long-term experiment with the same cell population [130].

In a subsequent 5-day study, the cells upregulated mitogen-activated protein kinase 1/2 (MAPK1/2) [131]. The authors observed upregulation of VIM protein and gene expression in both µ*g* cell populations, maybe because of an epithelial to mesenchymal transition. The *RHOA* gene was upregulated in both cell populations, perhaps because cells had to constantly regain cytoskeletal tension because of the loss of tension caused by µ*g*; however, the RhoA protein was not overexpressed. Finally, MAPK1 was overexpressed at the gene and protein levels in the µ*g* cell populations.

Bauer et al. [132] performed a semantic study on human breast cancer cells regarding posttranslational modifications of proteins in cells cultured in µ*g* conditions. They found that µ*g* influences the expression of adhesion proteins and enzymes for the sialylation of these adhesion proteins [132].

A study comparing the effects of s-µ*g* using an RPM on human breast cancer cells and normal cells showed an increase in apoptosis and altered cytoskeletal architecture in cells that form MCS in s-µ*g* as opposed to cells that adhere to the flask walls. There was increased AKT and ERK pathway activity, which had a protective effect, slowing down apoptosis [133]. Cancer and normal cells activate different pathways to resist apoptosis once the adhesion to the ECM is disturbed, but regardless of the activated pathway, the response is not sufficient to counteract the stress caused by loss of adhesion to the ECM, an essential survival signal for most cells.

A recent study with MCF-7 and MDA-MB-231 breast cancer cells cultured in an RPM for 24 h showed distinct AD and MCS populations and cytoskeletal alterations. Critical roles for E-cadherin, fibronectin, β-catenin, and vinculin were established as mediators for MCS formation in s-µ*g* [134].

Chen et al. [135] reported that dysregulated extracellular vesicle (EV) protein cargo derived from MDA-MB-231 cells in µ*g* is closely associated with GTPases and purine metabolism. This alteration in purine metabolism could be related to the altered metabolism of MCS. For example, transformed mammary epithelial cells grown as spheroids increase proline catabolism to sustain adenosine triphosphate (ATP) production by the mitochondria and, at the same time, to increase mitochondrial antioxidant power [136]. In another study, the authors used a serum-free medium, which is known to influence the extracellular vesicle assembly—for example, in neuroblastoma cells, it alters the quantity and protein composition of EV [137].

### 6.2. Thyroid Cancer

Thyroid cancer is the most abundant tumour of the endocrine system. Similar to breast cancer cells, thyroid cancer cells form MCS when exposed to µ*g*. Most of the studies are from FTC-133 cells, which derived from a metastasis of follicular thyroid cancer.

#### 6.2.1. Real Microgravity Studies

An analysis of FTC-133 follicular thyroid cancer cells cultured for 5 and 10 days on the ISS, vascular endothelial growth factor (VEGF) protein release was higher in the samples that experienced r-µ*g* compared with the RPM samples or 1 *g* control. *VEGFA* gene expression was downregulated in the r-µ*g* group but only minimally reduced in the s-µ*g* group. The higher release of VEGF could point towards a greater angiogenic potential when exposed to r-µ*g* [138].

Supernatants of these same cell lines cultured on the ISS for 12 days showed differences in the number of secreted exosomes and the subpopulation distribution regarding surface protein expression, notably the surface expression of tetraspanins. The tetraspanins CD63 and CD81 were upregulated in µ*g*, while CD9 remained unchanged. These proteins play a role in cell motility, cell proliferation, and adhesion, which indicates an alteration of these processes in µ*g* [139]. Recently, analyses of the exosomal microRNA composition [140] after several days of µ*g* elucidated some of the proteomic changes published earlier [141]. An array scan of 754 miRNA targets showed more than 100 differentially expressed miRNAs in the space-flown cells, many of which are involved in thyroid disorders [140].

In an experiment with short-term r-µ*g* exposure of the same cell line for 6 min through a sounding rocket, RNA was fixed before and after the µ*g* phase with RNA*later*. Some cells were exposed to hyper-*g* (18 *g*) to determine the effect of the hyper-*g* phase during the launch of the sounding rocket. Pathway analyses revealed central functions of VEGFA and endothelial growth factor (EGF). Hyper-*g* induced a significant upregulation of *TUBB1*, *VIM*, *RDX*, *CAV1*, *VEGFA*, and *BCL2* [142]. The moderate gene expression changes indicate the cells’ orbital survival. Quantitative polymerase chain reaction (qPCR) analyses revealed no remarkable expression changes in controls and hyper-*g* samples at the end of the first minute of launch. The samples exposed to hyper-*g* for 1 min presented moderate gene expression changes in *COL1A1*, *VCL*, *CFL1*, *PTK2*, *IL6*, *CXCL8*, and *MMP14*. Finally, samples incubated in an RPM indicated that µ*g* is a more potent regulator of gene expression than hyper-*g* [22].

A semantic analysis of posttranslational modifications of proteins, particularly those that showed significant accumulation in MCS compared with 1 *g* monolayer cells, revealed a total of 72 different classes of posttranslational modifications for these proteins, comprising mainly phosphorylation, glycosylation, ubiquitination, and acetylation [143].

#### 6.2.2. Simulated Microgravity Studies

Deep proteome analysis of FTC-133 cells cultured for different times before RPM exposure confirmed and explained the observations that factors inducing angiogenesis, the composition of integrins, the density of the cell monolayer, and enhanced production of caveolin-1 and NF-κB p65 play a role during MCS formation. FTC-133 cells that grow in monolayers or MCS after RPM exposure incorporate vinculin, paxillin, FAK1, and ADP-ribosylation factor 6 in different ways into the focal adhesion complexes [60].

Seeking to study MCS formation in FTC-133 cells, researchers used dexamethasone during a 3-day culture in an RPM. This drug dose-dependently inhibited MCS formation; this process involved the E-cadherin/β-catenin pathway. Wnt/β-catenin signalling and expression patterns of essential genes in cancer cell growth and survival (*NFKB2*, *VEGFA*, *CTGF*, *CAV1*, *BCL2(L1)* or *SNAI1*) were affected by dexamethasone. The NF-κB pathway was not influenced by dexamethasone, suggesting that this pathway may not mediate the MCS formation effects of this drug. The researchers proposed a more complex network regulating MCS formation [25].

Based on these studies, we can observe that DEX and PP2 may affect some similar pathways regarding MCS formation, as both inhibit its formation, even in different cell lines. These findings suggest essential targets for cancer metastasis therapies, such as the Src kinases.

### 6.3. Melanoma

BL6-10 melanoma cells cultured in a clinostat reduced proliferation, adhesion, and invasiveness in vitro and decreased tumour lung metastasis in vivo after injection in a mouse model. S-µ*g* reduces the formation of FA and activation of FAK and Rho family proteins (RhoA, Rac1, and Cdc42) and mammalian target of rapamycin complex 1 (mTORC1) but activates AMPK and ULK1 kinases [46]. It also inhibits NADH induction and glycolysis but induces mitochondrial biogenesis. The administration of a RhoA activator effectively counteracts the s-µ*g* alterations and effects on mitochondria biogenesis or glycolysis, and it also reverses the altered cell proliferation and tumour metastasis. The use of an mTORC1 inhibitor produces opposite responses and mimics s-µ*g* effects at normal gravity. All these findings fit into the model of µ*g* sensing presented above.

In a subsequent study, the researcher showed that s-µ*g* downregulates the expression of mTORC1-related Raptor, pS6K, pEIF4E, pNF-κB, and pNF-κB-regulated Bcl2, and induces relocalisation of pNF-κB from the nucleus to the cytoplasm. It also inhibits the nuclear envelope proteins lamin-A, emerin, sun1, and nesprin-3, which control nuclear positioning and suppress nuclear positioning–regulated pERK1/2 signalling. Again, the mTORC1 inhibitor enhances apoptosis in cells under 1g condition via the mTORC1/NF-κB pathway. Moreover, as mentioned previously, FAK/RhoA activator reduces apoptosis; restores the cytoskeleton, focal adhesions, nuclear envelope proteins, and nuclear positioning; and reverts all the above-mentioned s-µ*g*-mediated changes. The authors cleverly concluded that the FAK/RhoA regulatory network may be a new target for novel therapeutics for humans under spaceflight [144].

### 6.4. Haematological Disorders

Hodgkin lymphoma cells were exposed to a 3D clinostat for 2 days. They showed increased ROS production and NADPH oxidase family gene expression and decreased mitochondrial mass, ATPase, ATP synthase, and intracellular ATP levels. Moreover, this autophagy was inhibited by using a ROS scavenger. This effect was more pronounced in Hodgkin lymphoma cells with a high turnover, suggesting that high-turnover cells are more sensitive to s-µ*g* [145].

Using an RCCS and leukaemic and erythroleukemic cells for 48 h, leukaemic cells treated with daunorubicin show increased chemotactic migration after s-µ*g* compared with 1 *g* controls. Cells treated with doxorubicin showed enhanced migration both in 1 *g* and following μ*g*. The increase in ROS production in s-µ*g* was cell-type dependent, with acute myeloid leukaemia (AML) cells showing no increase, while chronic myeloid leukaemia (CML) cells have an increase in ROS production [146].

### 6.5. Gastrointestinal Tract and Liver

Colorectal cancer cells (DLD1, HCT116, and SW620) cultured in s-µ*g* in an RCCS-High Aspect Ratio Vessel for 48 h died via apoptosis. Gene expression in DLD1 cells showed upregulation of the tumour suppressors PTEN and FOXO3; this leads to AKT downregulation and apoptosis induction through upregulation of CDK inhibitors. In addition, there was elevated hypoxia and mitochondrial membrane potential in the cell clumps, leading to adaptive responses like morphogenetic changes, migration, and deregulated autophagy when placed in traditional culture [147].

In an exciting setup using HCT116 human colorectal cancer cells to compare a µ*g* method with a different 3D culture system (to distinguish the effects due to µ*g* from the ones due to 3D culture), in s-µ*g* there was a distinct CD133/CD44 dual-positive cell population compared with the 3D culture and 1 *g* control. Moreover, 3D culture and s-µ*g* increased autophagy and the number of individual giant cancer cells housing complete nuclear localisation of YAP were observed in s-µ*g* [17].

HGC-27 gastric cancer cells cultured in an RCCS for 3 days were analysed with liquid chromatography–mass spectrometry, and 67 differentially regulated metabolites were identified. Phosphatidylethanolamine, phosphatidylcholine, arachidonic acid, and sphinganine were upregulated in s-µ*g*. Sphingomyelin, phosphatidylserine, phosphatidic acid, L-proline, creatine, pantothenic acid, oxidised glutathione, adenosine diphosphate, and adenosine triphosphate were downregulated. Compound analysis revealed that lipids and lipid-like metabolites were primarily affected in the s-µ*g* environment [148].

The hepatoblastoma cell line HepG2 was cultured in a 3D clinostat (Gravite) for up to 3 days. Immunoblotting indicated that in µ*g*, CDDP-induced ATM/p53 signalling and caspase-3 was cleaved earlier. µ*g* decreased the expression of the p53 targets BAX and CDKN1A but increased the mRNA levels of *PTEN*, *DRAM1* (promotes apoptosis), and *PRKAA1* (promotes cell proliferation). It also decreased the levels of mTOR and increased the microtubule-associated protein light chain 3 (LC3)-II/I ratio, suggesting autophagy activation. CDDP-induced cleavage of caspase-3 was increased during the early phase in the microgravity group. Cleaved caspase-3 was seen in the CDDP treated µ*g* group even when using a p53 mutant with constitutive expression, indicating a p53 independent mechanism [149].

### 6.6. Prostate Cancer

Prostate cancer is the most prevalent cancer in men worldwide; few studies have been performed on µ*g* and prostate cancer. PC3 cells were cultured in an RPM for 3 or 5 days—most cells detached from the flask bottom and formed MCS. The 5-day gene expression results found significant downregulation of *VEGF* in AD and MCS populations; *FN1*, *CDH1*, and *LAMA3* in the AD population; and *SCR1* in the MCS population. There was also significant upregulation of *FLT1*, *AKT*, *ERK1*, *ERK2*, *LCN2*, *COL1A1*, *TUBB*, and *VCL* in the AD and MCS populations; increased *FLK1*, *FN1*, and *COL4A5* expression in the MCS population, and increased *LAMB2*, *CDH1*, *RAF1*, *MEK1*, *SRC1*, and *MTOR* expression in the AD population. Most importantly, VEGFA and NGAL protein secretion decreased. Cytoskeletal alterations (F-actin) were visible, as well as a deposition of collagen in MCS. The significant upregulation of genes belonging to the PI3K/AKT/mTOR (PAM) pathway indicated their involvement in cellular changes occurring in µ*g* [150].

### 6.7. Lung Cancer

Lung cancer is a leading cause of cancer-related mortality worldwide. Lung cancer is classified into two main types: small-cell lung carcinoma (SCLC) and non-small-cell lung carcinoma (NSCLC). Human lung cancer adenocarcinoma (A549) and squamous cell carcinoma (SCC and H1703) cells lines were grown in a 3D clinostat for 36 h. In SCC cells, the proliferation rate of the clinostat group was lower and the migratory ability was increased after exposure to µ*g* [151]. In the same cells, initial cell adhesion in µ*g* was low, but the normalised proliferation rate of A549 in s-µ*g* was higher than that in the 1 *g* controls. Wound healing results of A549 and H1703 showed rapid recovery in s-µ*g*. The migration rate of A549 was faster than that of H1703, both in the normal and low-proliferating conditions. Gene expression results showed that s-µ*g* accelerated the migration, where both highly expressed the migration-related genes *MMP2*, *MMP9*, *TIMP1*, and *TIMP2* compared with the control at 24 h. Moreover, MMP-2 protein synthesis indicated the weaker metastatic performance of H1703 compared with A549 cells [151,152].

In a study of the effects of s-µ*g* on SCC (CRL-5889) incubated in an RPM for 72 h, 3D MCS formation was observed. The actin filaments showed a shift in alignment from longitudinal to spherical, and the apoptotic rate was significantly increased in MCS compared with the 1 *g* control. *TP53*, *CDKN2A*, *PTEN*, and *RB1* gene expression was significantly upregulated in the AD cells in s-µ*g*, with an increase in the corresponding protein production of p14 and RB1 [15]. The researchers concluded that s-µ*g* alters cell adherence, increases the apoptotic rate of floating cells, and leads to upregulation of tumour suppressor genes in the AD cell population.

The A549 cell line (hypotriploid human alveolar basal epithelial), a lung adenocarcinoma model, was exposed to s-µ*g* for various time points, resulting in the generation of polynucleated cells, cell cycle imbalance, growth inhibition, and highly damaged mitochondria. Global miRNA analysis defined a pool of miRNAs associated with μ*g* exposure, mainly involved in cell cycle regulation, apoptosis, and stress response [153].

### 6.8. Brain Tumours

Glioma is the most common primary tumour in the central nervous system. A human glioma cell line (U251) was cultured on coverslips and then exposed to s-µ*g* in a two-dimensional (2D) clinostat for up to 3 days. S-µ*g* inhibited proliferation and induced apoptosis, while expression of the apoptosis-associated protein p21 was upregulated and IGFBP-2 was downregulated [154].

S-µ*g* induced apoptosis of U251 cells when cultured on the SM-31 random locator. The FAK/RhoA/Rock and FAK/Nek2 signalling pathways were attenuated, destabilising the actin cytoskeleton and centrosome disjunction. This caused G2/M cell cycle arrest and inhibition of cell viability and migration. Overexpressed FAK reversed the s-µ*g*-mediated inhibition of viability and migration, which increased downstream RhoA/Rock signalling and Nek2 expression [47].

### 6.9. Bone Tumours

The Ewing’s sarcoma cell line A673 was incubated in an RPM for 24 h, resulting in AD and MCS populations. *EWS*/*FLI1* gene expression was upregulated in the AD and MCS populations compared with the 1 *g* control, and *CXCR4* and *CD44* expression was increased only in the MCS population. *CAV1* was upregulated and *DKK2* and *VEGFA* were downregulated in both populations. EWS/FLI1 protein was elevated only in the AD population, but CD44 protein decreased in the MCS and AD populations. The inhibition of CXCR4 did not change the spheroid count or structure [155].

Table 1 provides an overview of the studies discussed above.

## 7. Conclusions and Future Perspectives

Epidemiological data have shown that the compound effect of radiation and µ*g* increases cancer risk, even considering the healthy worker effect. In most cases, cancer cells in weightlessness show reversal of malignancy and the induction of apoptosis. However, these findings do not mean that this tendency would be the same at the tissue or organ level. Sending a cancer patient to space will probably not be a cure for cancer because most of the observed effects happen at the single-cell culture level and seem to come from the interactions of the cell with its environment. Cancer studies of cells in µ*g* help cancer biologists to explain cancer cell features that are exhibited only in the unloading conditions and can confirm the current paradigms that the ECM and surrounding microenvironment are vital for cancer cell malignancy and survival. We have highlighted the importance of cancer studies in µ*g*, which has undoubtedly helped us discover some cancer cell vulnerabilities that would be impossible to note in normal gravity conditions. For example, the changes in molecules involved in cell adhesion that are very commonly observed in space conditions, like changes in E-cadherin, ICAM-1, VCAM-1, and CD44 protein synthesis; also important to mention is the finding that depending on if cells are growing as monolayers or MCS after RPM exposure, cells incorporate vinculin, paxillin, FAK1, in different ways into the focal adhesion complex. Even more exciting findings will appear by applying the current knowledge of mechanobiology to µ*g* research and finding the mechanobiological Achilles heel of cancer cells.

## Figures and Tables

**Table 1:** A summary of the most important recent findings.

Cancer Type	Microgravity Effects	s-µ*g*	r-µ*g*
Breast	NF-κB p65 plays a crucial role in MCS formation [128]	RPM	
	Decreased E-cadherin in MCS; the balance of proteins that up- or downregulate E-cadherin mediates the tendency to form MCS [26]	RPM	
	Upregulation of *KRT8*, *RDX*, *TIMP1*, *CXCL8* mRNAs and downregulation of *VCL.* E-cadherin protein was significantly reduced [124]; rearrangement of F-actin and tubulin, with the formation of holes		SR & PF
	MCS have an altered cytoskeleton and appreciable apoptosis after 72 h; survival strategies cannot provide sufficient protection [133]	RPM	
	The process of linking cells to each other or the ECM under µ*g* includes sialylation of extracellular domains of adhesion proteins [132]	RPM	ISS
	Vinculin and β-catenin are critical to form MCS during incubation in an RPM for 24 h [134]	RPM	
	MCS formation; *BRCA1* increased, *KRAS* decreased in AD cells; *VCAM1* upregulated, *VIM* downregulated in µ*g* [130]	RPM	
	Increased metastatic ability; considerable changes in morphology, cytoskeletal shape, and gene expression [131]	RPM	
	Induces gene expression of cell adhesion molecules [125]	RPM	PF
	EV release rate decreases while average EV size increases; significant correlation with GTPases and proliferation [135]	Gravite	
	Lysosomal vesicles, cyclin D3, and apoptosis increase; migration ability and the expression of BCL-2 and MMP9 proteins decrease [129]	RWV	
Thyroid	Altered integrin signalling, facilitating cytoskeletal changes, and weakening focal adhesion complexes, promoting MCS formation [60]	RPM	
	Moderate gene expression changes indicate orbital survival [142]	hyper-*g*	SR
	µ*g* is a more potent regulator of gene expression than hyper-*g* [22]	RPM	SR
	Proteins undergo extensive posttranslational modification [143]	s-µ*g*	
	Spheroids formed in all hardware units; enhanced release of VEGF versus RPM samples [138]	RPM	ISS
	Alters expression of adhesion proteins and enzymes for their posttranslational modifications [132]	RPM	
	Dexamethasone inhibits the formation of MCS in a dose-dependent manner through the E-cadherin/β-catenin pathway [25]	RPM	
	Differences in the number of secreted exosomes, alteration of their population regarding the tetraspanin surface expression [139]		ISS
Skin (melanoma)	Inhibits focal adhesions, leading to reduced proliferation and metastasis via FAK/RhoA-regulated mTORC1 and AMPK pathways [46]	Clinostat	
	Fewer focal adhesions; enhanced apoptosis via FAK/RhoA-mediated mTORC1/NF-κB and ERK1/2 pathways suppression [144]	Clinostat	
Haematological	Induced autophagy via mitochondrial dysfunction [145]	3D-C	
	Modulated chemotherapeutics effects on cancer cell migration [146]	RWV	
Gastrointestinal	PTEN/FOXO3/AKT pathway regulates cell death and mediates morphogenetic differentiation [147]	RCCS-H	
	More polyploid giant cancer cells and YAP nuclear localisation [17]	RCCS	
	Effects on lipid metabolism [148]	RCCS	
	Enhances CDDP-induced apoptosis via independent of p53 [149]	RPM	
Prostate	Influenced VEGF, MAPK, and PAM signalling [150].	RPM	
Lung	Cell type–dependent effects on proliferation and migration [151]	3D-C	
	Promotes migration of non-small cell lung cancer [152]	RPM	
	Apoptosis induction and alteration of cell adherence [15]	RPM	
	Mitochondria are susceptible to μ*g*; global miRNA analysis defined a pool of miRNAs associated with μ*g* exposure [153]	RPM	
Brain	Influence on proliferation and apoptosis in glioma cells [154]	2D-C	
	Inhibits viability and migration via FAK/RhoA/Rock and FAK/Nek2 [47]	SM-31	
Bone	Increased EWS/FLI1 expression; CXCR4 does not affect MCS formation [155]	RPM	

Abbreviations: 2D-C: two-dimensional clinostat; 3D-C: 3D clinostat; AD: adherent population; CDDP: cis-diamminedichloroplatinum; ECM: extracellular matrix; ES: Ewing’s sarcoma; EV: extracellular vesicle; H: high aspect ratio vessel (HARV); hyper-*g*: hypergravity; ISS: International Space Station; MAPK: mitogen-activated protein kinase; MCS: multicellular spheroids; µ*g*: microgravity; NF-κB: nuclear factor kappa B; PAM: PI3K/AKT/mTOR; PF: parabolic flight; RCCS: rotary cell culture system; RWV: rotating well vessel; SM-31: random locator developed by the Center for Space Science and Applied Research, Chinese Academy of Sciences; SR: sounding rocket; VEGF: vascular endothelial growth factor.

## Data Availability

Not applicable.
